# Prostate Cancer Tumor Stroma: Responsibility in Tumor Biology, Diagnosis and Treatment

**DOI:** 10.3390/cancers14184412

**Published:** 2022-09-11

**Authors:** Luis O. González, Noemi Eiro, Maria Fraile, Nana Beridze, Andres R. Escaf, Safwan Escaf, Jesús M. Fernández-Gómez, Francisco J. Vizoso

**Affiliations:** 1 Department of Anatomical Pathology, Fundación Hospital de Jove, Avda. Eduardo Castro, 161, 33920 Gijón, Spain; 2 Research Unit, Fundación Hospital de Jove, Avda. Eduardo Castro, 161, 33920 Gijón, Spain; 3 Department of Urology, Hospital Universitario Central de Asturias, Universidad de Oviedo, Avda. de Roma s/n., 33011 Oviedo, Spain; 4 Department of Surgery, Fundación Hospital de Jove, Avda. Eduardo Castro, 161, 33920 Gijón, Spain

**Keywords:** cancer-associated fibroblasts, mesenchymal stromal cells, tumor microenvironment, extracellular vesicles, exosomes, biochemical recurrence in prostate cancer

## Abstract

**Simple Summary:**

The crosstalk between prostate stroma and its epithelium is essential to tissue homeostasis. Likewise, reciprocal signaling between tumor cells and the stromal compartment is required in tumor progression to facilitate or stimulate key processes such as cell proliferation and invasion. The aim of the present work was to review the current state of knowledge on the significance of tumor stroma in the genesis, progression and therapeutic response of prostate carcinoma. Additionally, we addressed the future therapeutic opportunities.

**Abstract:**

Prostate cancer (PCa) is a common cancer among males globally, and its occurrence is growing worldwide. Clinical decisions about the combination of therapies are becoming highly relevant. However, this is a heterogeneous disease, ranging widely in prognosis. Therefore, new approaches are needed based on tumor biology, from which further prognostic assessments can be established and complementary strategies can be identified. The knowledge of both the morphological structure and functional biology of the PCa stroma compartment can provide new diagnostic, prognostic or therapeutic possibilities. In the present review, we analyzed the aspects related to the tumor stromal component (both acellular and cellular) in PCa, their influence on tumor behavior and the therapeutic response and their consideration as a new therapeutic target.

## 1. Introduction

The prostate and breasts are accessory sexual glands present only in mammals. Cancers of these origins are major health issues of the new century worldwide. Breast cancer accounts for over 25% of women’s cancers universally, implying a high risk (more than 10%) of developing this cancer during a woman’s lifetime [1]. Prostate cancer (PCa) is a common cancer among males [2]. In addition, the number of men diagnosed with PCa is growing all over the world [3,4]. On the other hand, 30–40% of patients with PCa experience biochemical recurrence (BCR) after radical prostatectomy (RP), and approximately 26–30% of these will develop into advanced and metastatic disease within the next five years [5,6]. In this respect, clinical decisions about adjuvant therapy based on a combination of androgen deprivation therapies (ADT) with radiotherapy, chemotherapy and/or immunotherapy [7,8] are important. Furthermore, PCa may display resistance to ADT, which is often accompanied by the occurrence of metastasis [9] and related deaths within 2–4 years [10].

Currently, the prognostic factors established for PCa are the TNM Classification of Malignant Tumors, the surgical margin status, the PSA (prostate-specific antigen) serum level and Gleason’s score. The combination of clinical staging and Gleason score remains the best predictor of a prognosis. However, although the Gleason classification is the most widely used, it persists in being deficient to elucidate the tumor behavior [11]. For all of this, new approaches to tumor biology are required, from which further prognostic evaluations and complementary strategies may be appointed.

**Figure 1:**
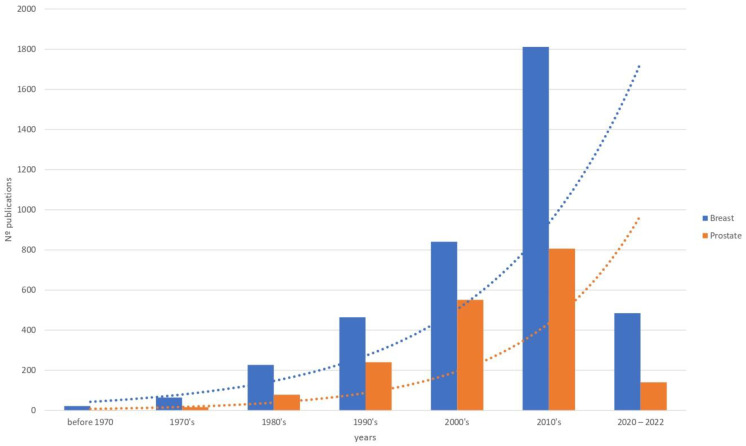
Evolution of the number of published studies based on the tumoral stroma in breast and prostate carcinomas. Source: PubMed.

Since the 1950s, some studies have supported the hypothesis of biological, genetic and epidemiological similarities between breast cancer and PCa [12,13]. Nevertheless, it is striking that while many studies have been published on the biological implication of the tumor stroma of breast cancer, little research exists on the same aspects in PCa. Figure 1 illustrates the changes in published studies of the stroma in breast cancer and PCa. As can be seen, there are approximately twice as many such works on breast cancer.

In this review, we discuss the relevant aspects of the complex tumor/stroma relationship in PCa, with its possible prognostic and potential therapeutic implications.

## 2. Normal Prostatic Stroma, Reactive Stroma in Benign Pathologies and in Preneoplastic Lesions

**Figure 2:**
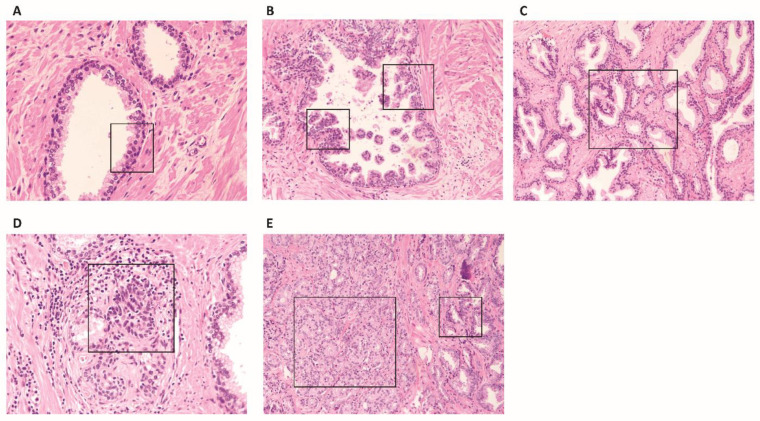
Representative tissue section. (**A**) Normal prostate tissue with epithelial luminal and basal layers and the surrounding stroma tissue (200×). (**B**) Benign prostate hypertrophy (BPH) tissue showing cell proliferation and migration (200×). (**C**) Proliferative inflammatory atrophy (PIA) tissue (100×). (**D**) High-grade prostatic intraepithelial neoplasia (HGPIN) in the peripheral zone of the prostate (200×). (**E**) Prostate cancer tissue (100×).

The prostate tissular architecture closely resembles that of the breasts in that it also comprises ducts with epithelial luminal and basal layers and the surrounding stroma tissue (Figure 2A). In normal prostatic tissue, the epithelium and stroma interact to maintain the physiological homeostasis. The stromal compartment is composed of a collagen-rich extracellular matrix (ECM) and cells. The ECM contributes to the establishment, separation and preservation of differentiated tissues. In addition, the ECM has influence on physiological signaling, since cells interact with the ECM by expressing receptors at their cell surfaces [13]. The basement membrane (BM) is an ECM structure that separates the epithelium from the stroma, and it is implicated in tissue resistance [14]. The ECM is configured with structural proteins (elastin and fibronectin), fibrillary proteins (collagens) and hydrated gel-forming macromolecules (proteoglycans or hyaluronic acid) [15]. Nevertheless, the ECM composition can vary considerably according to the tissue type.

The cells components from the stroma include fibroblasts, smooth muscle cells and immune cells, blood vessels and nerves. The crosstalk between epithelial cells and all the adjacent stromal cells is key to preserve the homeostasis in the normal prostatic tissue [16]. Thus, for example, it is known that prostatic androgen-stimulated smooth muscle cells induce the correct differentiation of epithelial cells through the release of regulatory molecular factors [17]. However, the homeostatic regulation capacity of the stroma in the face of tissue injuries or microbial infections can be reduced with the processes associated with aging. In these circumstances, the stromal cells can secrete proinflammatory cytokines (such as CXCL12 and CXCL5) that induce a proliferative activity of the epithelium and cause benign prostate hypertrophy (BPH) [18].

The deregulation of epithelial–stromal interactions is not only considered to be responsible for initiating and/or promoting proliferative diseases such as BPH but also PCa [19,20]. BPH typically happens in the 80–90% of older men in their 70s [21]. The prostate epithelium of BPH maintains its structural organization but characteristically consists of a highly proliferative epithelium, which results in enlarged nodules surrounding the stroma, showing the typical features of fibrotic diseases and reactive stroma with proinflammatory properties [22]. The BPH stroma is primarily composed of proliferating fibroblasts and myofibroblasts secreting ECM proteins, such as collagen type I and tenascin C [22]. Tenascin C is a glycoprotein involved in tissue remodeling such as cell adhesion and migration [23] (Figure 2B).

The morphological and functional changes in stroma progression to premalignant lesions are relevant. Focal atrophic lesions usually occur in the peripheral zone of the prostate [24] and are characterized by enhanced cell proliferation [25]. These types of lesions, characterized by chronic inflammation, are termed proliferative inflammatory atrophy (PIA) (Figure 2C) and can be due to several causes, such as diet type, cell damage (e.g., chemical exposure), infectious agents, hormonal changes or urinary retention [24]. Instead, PIA lesions could be precursors to high-grade prostatic intraepithelial neoplasia (HGPIN) in the peripheral zone (Figure 2D), which may subsequently progress to invasive PCa [24] (Figure 2E). This is attributable to stromal transformations, which lead to starting a tumorigenic process, such as an increase in oxidative stress. Therefore, it is known that, in inflammatory lesions, there is both protumor genomic instability and modifications in the gene expression, which are, in part, provoked due to macrophages releasing reactive oxygen species (ROS) and reactive nitrogen species [24,26]. Interestingly, this stromal transformation is considered to be the preliminary stage of HGPIN and PCa [27]. It was also observed that fibroblasts adjacent to the HGPIN foci are induced to acquire the cancer-associated fibroblasts (CAFs) phenotype via secreted factors by prostate epithelial cells, such as kallikrein-related peptidase-4 (KLK4) [28]. As a result, all these data suggest that induced changes in the stromal component of the prostatic tissue contribute to the progression to invasive PCa.

## 3. Reactive Stroma in Cancer

Homeostasis derived from a constant and self-regulated epithelial–stromal interaction is definitely disrupted in carcinomas, generating a tumor microenvironment (TME) that promotes tumor progression [29]. In 1986, Dvorak described tumors as “non-healing wounds” and suggested that stromal cells actively interact with epithelial cancerous cells [30]. In this stage, stromal cells often react with a fibrotic reaction around tumors [31].

The term “reactive stroma” consists of a set of alterations in the TME as a reaction to the presence of tumor cells due to an altered ECM deposition, neovascularization and the increased presence of myofibroblast-like CAFs and immune cell infiltration [32]. This concept posits that cancer cells cannot promote the disease by themselves but might recruit and modulate resident cell types to cooperate to promote tumor progression [33,34]. It was even stressed that the presence of a modified TME may be sufficient to promote epithelial cell tumorigenesis, even without genetic alterations [34]. Its influence is such that, if a normal microenvironment is restored, cancer cells may lose their tumorigenic phenotypes and capabilities [33,34].

The composition of the tumor stroma varies across different tumor types and within the same tumor type. The tumor stroma consists of ECM, the nonmalignant cells of the tumor mass and their cell components [35].

### 3.1. Tumoral Stroma ECM

First, the ECM forms a physical barrier, specially represented by the BM, which is more compact than the interstitial matrix, preventing the invasion of cancer cells and subsequently providing protection [36]. Hence, the interaction between cancer cells and the ECM is the first and key dynamic process in tumor pathobiology [14,37]. This remodeling process is perturbed during cancer with an abnormal ECM deposit, leading to stiffness and tumor progression [37]. In this context, enzymes secreted by tumor cells, such as lysyl oxidases (LOX), are capable of crosslinking collagen and, thus, to building up one collagen I structure that promotes metastasis [32,38,39]. In addition to its structural function, the ECM represents a reservoir for bioactive molecules that may positively impact on several biological basic processes related with tumor progression [14].

### 3.2. Cell Components of Tumoral Stroma

**Figure 3:**
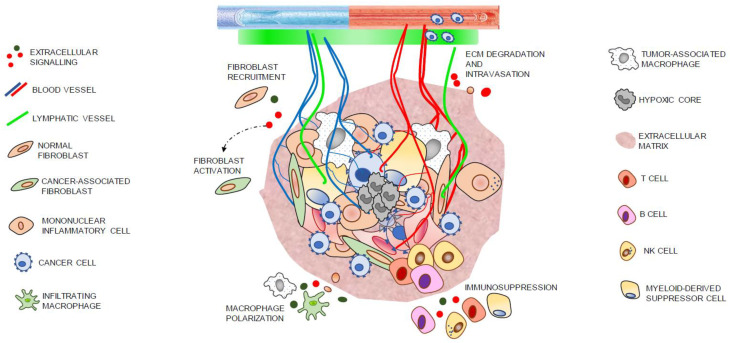
Schematic representation of the cellular components from a prostate tumor.

Different cell types play a role in tumor–stroma interactions. These ones include resident cells, such as CAFs, endothelial cells and pericytes, neural crest cells and mesenchymal stromal/stem cells (MSC). No resident stromal cells comprise immune cells, which infiltrate solid tumors (Figure 3).

#### 3.2.1. Myoepithelial Cells and Fibroblasts

The cell components of the ECM, such as myoepithelial cells or fibroblasts, are characterized by predominantly performing protective functions to inhibit tumor progression. In breast cancer, the disruption of myoepithelial cells appears to be a cardinal milestone in tumor progression. The myoepithelial cells are situated between the stroma and the luminal cells, creating a natural separation between both morphological structures [40,41,42]. In addition, myoepithelial cells reduce the gene expression of MMP-2, MMP-9 and MT1-MMP, thereby reducing cancer cells’ invasive capacities [43]. Myoepithelial cells also inhibit angiogenesis by expressing MMP inhibitor TIMP-1, thrombospondin-1 and bFGF receptors [44,45]. In addition, myoepithelial cells, by expressing high levels of fibronectin, laminin and collagen [46,47], also participate in the accumulation of ECM and BM rather than degrading it. Therefore, all of these data suggest that myoepithelial cells can have multiple positive roles in preventing tumorigenesis.

On the other hand, it has been suggested that stromal fibroblasts might have a protective task involving cancer. Thus, it has been reported that these stromal cells may decrease EMT, invasion and metastasis by secreting factors such as caveolin-1, podoplanin [48], SLIT2 and asporin [49]. Nevertheless, despite all of these protective actions against tumor progression, stromal cells are mainly recognized by their protumor actions.

#### 3.2.2. CAFs and Tumor Progression

Cancer cells secrete cytokines and chemokines, such as TGF-β, involved in CAF recruitment and activation [50,51]. In fact, CAFs represent the most plentiful stromal component in PCa. CAFs are described as spindle-shaped cells but, compared to normal fibroblasts, may be identified by the overexpression of molecular markers such as α-SMA, fibroblast activation protein (FAP), PDGFR-β or fibroblast-specific protein-1 (FSP-1) [52].

Resident fibroblasts can be the source of the CAF population. In this sense, it has recently been reported how Yes-associated protein 1 (YAP1) can convert normal fibroblasts into CAFs in the PCa TME. In addition, silencing YAP1 in tumor stromal cells can inhibit tumor growth in PCa [53]. However, CAFs can also originate from other sources, including MSC, epithelial cells, pericytes, adipocytes and endothelial cells [54]. Consequently, in PCa, the existence of different populations of CAFs has been described, which probably reflects the different cell origins of CAFs. CAFs with high CD90 levels of expression exhibited an increased proportion of numerous genes associated with tumor progress, including TGF-β, the angiogenic factors vascular endothelial growth factor (VEGF) and fibroblast growth factor 2 (FGF2) and the cytokines interleukin (IL)-6 and chemokine (C-X-C motif) ligand 12. In addition, the coexistence of subpopulations of CAFs that do not express and others that express TGF-β receptor II (TGFβRII) has been identified, which seems to contribute to tumor progression and evidence of the heterogeneity of CAFs in PCa [55,56].

Several studies have identified CAFs as promoting tumor cell growth, invasion, epithelial-to-mesenchymal transition (EMT) and ADT resistance in PCa [35]. CAFs may positively influence tumor invasion through indirect or direct actions, such as cell-to-cell contact, which contribute to the regulation of the cancer cell motility through the modulation of Eph-Ephrin signaling [57]. It has also been suggested that direct contact between PCa cells and CAFs enhances tumor growth by activating Notch signaling in stromal cells [58].

CAFs can also influence tumor invasion trough indirect actions such as the overproduction of ECM components (collagen, tenascin-C, fibronectin or hyaluronate) [55,59,60,61] that favor tumor cell proliferation and invasion, leading to metastasis [15,62]. Indeed, it has been described that fibronectin produced by CAFs can establish a fiber-oriented network allowing migration pathways to cancer cells [61]. CAF contractile forces may alter the organization and the physical properties of the BM, making it permissive to tumor invasion [63]. However, there are a lot of data indicating a more complex role of CAFs in tumor progression.

CAFs induce ECM remodeling by secreting matrix proteases such as MMPs and FAP [64]. Especially relevant seems MMPs, secreted by both stromal and cancer cells, which are regulated by tissue inhibitors of matrix metalloproteinases (TIMPs) [65,66]. During PCa development, the overexpression of MMP-1, -2, -7, -9 and -14 was found in stroma and circulation (Gong et al., 2014) [67,68], as well as an imbalance between MMPs and TIMPs, which enhances PCa cell invasiveness [66,69]. It has been proposed that the loss of Dickkopf-3 (DKK3) expression, a secreted protein that inhibits TGF-β signaling activity in both prostate epithelial and stromal cells, could explain the increased expression of MMPs in PCa. In addition, DKK3 silencing is associated with an increase release of MMP-2 and MMP-9 [70,71].

There are other mechanisms for which MMP activity also promote other key aspects of tumor progression, such as cell proliferation, apoptosis and angiogenesis [65,66], and the cleavage of growth factors with known tumorigenic properties, such as FGFs, TGF-β and HGF [72]. In turn, MMPs cause a clear EMT in cancer cells, as well as increased tumor growth and the development of metastases [73].

CAFs construct a metabolic symbiosis with PCa cells, bestowing cancer aggressiveness through a lactate shuttle. A crucial role of tumor mitochondria as a sensor and energy transducer of CAF-dependent metabolic reprogramming has been revealed. This underscores the dependence of cancer cells on CAF catabolic activity and mitochondria exchange [74]. Its activation relates to the reverse Warburg effect, a phenomenon occurring in several tumors, including PCa, in which CAFs performed aerobic glycolysis and provide lactate, as well as amino acids such as glutamine and ketone bodies, to oxidative tumor cells, which are able to use these nutrients as an energy source or incorporate them as metabolic precursors necessary for tumor development [75]. This shows that tumor cells depend, to some extent, on the stroma to maintain their metabolism and growth. CAFs have also been shown to improve immunosuppression in the TME partly through cytokine secretion, such as TGF-β and CXCL12, but equally across the expression of inhibitory molecules such as PD-L1. Moreover, CAFs can also promote the angiogenesis process by secreting factors such as VEGF-A, FGF2, PDGFC and CXCL12. There is another interesting mechanism lately described by which CAFs may protect cancer cells as they progress towards metastasis. Duda et al. indicated that CAFs can migrate with circulating tumor cells (CTCs) as wandering cell clusters. This mass migration unit boosts tumor cell survival and colonization in far-flung organs [76]. So much so that there is a correlation between the number of CAFs and disease progression in breast, colon and prostate cancer [77]. These results suggest the use of heterotypic clusters of CTCs and CAFs as potential markers of cancer progression, as well as potential targets in metastatic disease [78].

#### 3.2.3. CAFs and Therapy Response

CAFs can not only promote cancer progression but also its survival by creating a “protective niche” that keeps tumor cells alive by inducing a resistance to cancer therapy. There are several mechanisms by which CAFs may influence the efficacy of chemotherapeutic drugs. These stromal cells regulate the interstitial fluid pressure in the TME and therefore affect drug transport from the vasculature to tumor interstitium. In this regard, CAFs could reduce drug accessibility, especially at the center of the tumor [79]. In addition, various mechanisms were described by which CAFs can induce a resistance to different chemotherapeutic agents in PCa. There are data indicating that CAFs producing IL-6 inhibit doxorubicin-induced cell death by inhibiting p53 induction in PCa cells [80] but also through the release of glutathione, which decreases the ROS levels and avoids drug accumulation in cancer cells [81]. CAF-derived exosomes carrying miR-423-5p can also increase the resistance of PCa to taxane by blocking GREM2 through the TGF-β pathway [82].

Several studies found that CAFs are also active in promoting PCa resistance to antiandrogen therapies. AR indirectly inhibits the expression of inflammatory cytokines by CAFs such as CCL2 and CXCL8, known to promote PCa cell motility. CAFs secrete IL-6, which may activate AR transcriptional activity in PCa cells by modulating PI3K/AKT, MAPK and STAT3 signaling in the absence of androgens [83,84]. In a multivariate analysis, fibroblasts were the most significant cell type in determining the prognosis in PCa and associated with castration-resistant prostate cancer (CRPC) [85].

It has been described that ADT stimulates the proliferation of a subpopulation of CAFs, characterized by the expression of CD105, and produces frizzled-related protein 1 (SFRP1), a member of the Wnt signaling pathway, which supports the neuroendocrine differentiation of the adjacent epithelial cells [86]. This seems to indicate that CAFs participate in their progression towards neuroendocrine CRPC [86,87].

Interestingly, we found that one CAF population from PCa presented a higher expression of IL-6, FGF7, MMP2 and MMP11, with a lower expression of FGF10 and IL-17RB than normal prostatic fibroblasts [88], which was consistent with those found in breast cancer [89]. In addition, we also found that, at the same time, FGF7 is primarily expressed in CAFs from localized tumors, whereas MMP-11 and AR are overexpressed in CAFs from metastatic CRPC [88].

### 3.3. Immune Cells

Inflammatory cascades during PCa tumorigenesis have been extensively discussed [90,91]. The inflammatory process in the prostate gland may be caused by pathogens such as Chlamydia Trachomatis and Neisseria Gonorrhea, or noninfective shooters characterized by diet, urinary reflux or autoimmune processes [92]. Clinical studies detected an increased risk of PCa in men who had experienced infectious prostatitis [90]. Chronic inflammation in normal prostate tissue was evidently related to high-grade prostatic malignant tumors demonstrated by a biopsy [93]. In this setting, chronic inflammation transforms the prostatic microenvironment into a medium rich in immune cells, growth factors and chemokines and in proinflammatory cytokines, concomitantly interacting between them and with epithelial cells to induce proliferation and angiogenesis [94].

Immune cells can be recruited to the tumor by cytokines and chemokines such as CCL2 produced by cancer cells and CAFs [95,96,97]. Tumor-infiltrating leukocytes have been considered as part of the defense mechanism against tumor development [98] and also, in the end, interpreted as a failed attempt by the immune system to refuse the tumor. Instead, currently, it is common knowledge that leukocyte infiltration can promote tumor expansion, angiogenesis and tumor cell encroachment [99,100] due to the secretion of growth factors, proteases, chemokines and cytokines [101,102].

The immune cell infiltrate of tumors (Figure 3) comprises T and B cells, neutrophils and macrophages, among others [99]. Tumor-associated macrophages (TAMs) can exhibit a classically activated (M1) or an alternatively activated (M2) phenotype, defined as a tumor-suppressing or tumor-promoting phenotype, respectively, where the M2 phenotype is related to a worse prognosis [99,103,104]. This is evidenced by a study in which it was shown that TAMs are preferentially polarized as M1-like in colorectal cancer, as opposed to PCa, where TAMs are predominantly M2-like [105]. Likewise, it was reported that the presence of M2 within the TME from PCa is an independent predictor of extracapsular extension [106]. Nevertheless, myeloid-derived suppressor cells (MDSCs) are a heterogeneous population of immature myeloid cells with powerful immunosuppressing activity. MDSCs are classified as polymorphonuclear (PMN-MDSCs) or monocytic (M-MDSCs). PMN-MDSCs infiltrate much more easily into the stromal area than into the epithelial area of the tumor regions, and these stromal cell infiltrates were associated with vascularization in PCa [107]. In contrast, stromal T- and B-lymphocytes contribute to an immunological response that reduces cancer development and progression [108,109,110,111,112,113,114,115,116].

Some studies have attempted to ponder the impact of heterogeneity of the inflammatory component of the stroma on the PCa prognosis. The Cancer Genome Atlas (TCGA) database has provided a set of global gene expression profiles and clinical data on patients worldwide [117]. In addition, the ESTIMATE algorithm was developed to evaluate the expression levels of certain molecular entities in stromal and immune cells of the TME [118]. The immune-activated subtype, characterized by the activation of WNT/TGF-β, TGF-β1 and C-ECM signatures, is present in 14.9–24.3% of patients, which was associated with a good prognosis and a good response to anti-PD-1/PD-L1 therapy. ESTIMATE appears as a novel immune molecular classifier significantly associated with clinical prognosis and provides an innovative perspective on immunotherapeutic strategies for PCa patients [119]. In another, similar study, Zhao et al. found that eight individual differentially expressed genes (DEGs): C6, C7, S100A12, PAX5, FAM162B, MLC1, TCEAL5 and CAMK1G significantly predicted a favorable global survival, and one DEG, EPYC, was associated with immune cell infiltration, immune responses and a low overall survival [120].

The protumor effect of immune cells is mainly transmitted through cytokines. They may contribute to the creation of free radicals that can damage DNA, possibly causing mutations that lead to tumor formation, boosting cell proliferation and reducing apoptosis, stimulating EMT and angiogenesis or permitting tumor cell scape from immune surveillance. In contrast, cytokines can adjust an antitumoral response that seems to be dependent on the balance between pro- and anti-inflammatory cytokines [121] and the stage of tumor development [122]. Inflammatory cells generate high levels of proinflammatory cytokines and chemokines, such as IL-1β, IL-6, IL-8, IL-17, NF-κB, interferon-γ, VEGF and TNF-α. Some of these have been attached to tumorigenesis and prognosis in PCa [123]. High serum levels of TNF were reported in PCa hormone refractory conditions denoting an auspicious feature as a biomarker for CRPC. Elevated concentrations of NF-κB in the PCa microenvironment [124] alter the expression of cell cycle scriptwriters such as c-myc and cyclin-D1 and increases the expression of angiogenic factors, including IL-6, IL-8 and VEGF [125]. IL-6 was outlined as a key driver in PCa pathogenesis. As several studies demonstrated, increased serum levels of IL-6 match with metastatic or hormone-resistant PCa [126,127,128]. In addition, it was reported that targeting IL-6 with Siltuximab improved the disease outcomes in patients with metastatic refractory CRPC to the standard treatment [129]. On the other hand, there are also data showing that increased IL-8 concentrations within the PCa microenvironment increased cancer cell adherence to the endothelium, thereby improving tumor angiogenesis and metastatic propagation [130], as well as in docetaxel-refractory metastatic CRPC [130]. As a result, agents reducing the IL-8 levels such as naphthylamide help it deal with advanced forms of this malignancy [131].

### 3.4. Endothelial Cells

Endothelial cells (ECs) are pervasive within tumors and required for vessel development and function, particularly blood vessels, vital for providing oxygen and nutrients for tumor growth. The endothelial barrier keeps vascular and tissue homeostasis, and its dysfunction induces vascular permeability, which favors angiogenesis, inflammatory cell infiltration and tumor cell extravasation. Additionally, ECs can impact tumor progression through the secretion of several factors [132,133,134,135], induced by the crosstalk between a tumor and ECs [136]. The phenotypes of ECs differ depending on the tumors, as ECs from highly metastatic tumors exhibit a more proangiogenic phenotype than ECs from low metastatic tumors [137].

Tumor vascularization is characterized by the formation of immature blood vessels that fail covering pericytes [138]. The interaction between tumor cells and the surrounding stromal endothelial cells encourages an “angiogenic shift” by enhancing the proangiogenic factors such as VEGF. Zhao et al. evidenced that ECs are a substantial component of the TME for their contribution to boosting metastatic activity via silencing AR expression and transcriptional activity; therefore, their inhibition could impede PCa progression [120].

On the other hand, studying the phenotype of epithelial cells provides a clearer picture of the prognostic value of the tumor stroma. For example, for breast cancer, MMP-11 expression by ECs was associated with a shorter relapse-free survival, whereas TIMP-3 expression was linked to the small appearance of distant metastasis. Simultaneously, MMP-11 and TIMP-2 expression by ECs was associated with shorter global survival, whereas TIMP-3 expression by ECs was associated with an increased overall survival [139]. These results indicate that a strong MMP/TIMP expression by ECs from breast carcinomas can be due to interactions signaling between tumor cells and their surrounding microenvironment. Similar associations integrating morphology and biology should be explored in PCa.

### 3.5. Mesenchymal Stromal Cells

It is widely accepted that PCa originates from cancer stem cells (CSCs). Albeit prostate CSCs constitute a smaller percentage of the total tumor mass, there are data pointing out that they have several mechanisms related with PCa progression, such as improved DNA repair, antioxidative stress, autophagy, the initiation of antiapoptotic signaling, resistance to therapy, including radiotherapy, or EMT [140,141].

MSC are also part of the PCa tumor stroma and promote its progression. Essentially, MSC are adult multipotent stromal cells characterized by the expression of surface markers (CD73, CD90 and CD105), with the capability of self-regeneration and differentiation into osteoblasts, chondrocytes and adipocytes [142,143]. In physiological conditions, MSCs interact with the surrounding cells by secreting soluble factors, such as cytokines and growth factors, therefore contributing to tissue homeostasis and immunoregulation. However, MSCs also bear a relevant role in the tumor–stroma crosstalk [144,145]. MSCs can be recruited by neoplastic cells to the tumor site employing chemotactic factors such as MMPs, inflammatory cytokines and growth factors [146]. These steam cells have also shown several protumor behaviors, such as increasing the tumor growth speed [147] and angiogenesis [148] and onset EMT [149], along with modification of the extracellular matrix [150], in order to bolster the migration and implantation of metastasis [151]. MSCs prompt the suppression of immune effector cells [152,153], as well as the expansion of the immune regulatory ones [153,154], thus developing resistances to cancer therapies [155,156]. Specifically, in the TME, besides the MSCs being a source of CAFs, they may be able to transdifferentiate into MDSCs or M2-type macrophages under the influence of cytokines or chemokines [157].

In relation to PCa, there are data that supports the cooperation of CSCs and mesenchymal cells in metastasis development and hormone resistance [158]. Thus, a novel interaction between MSC and PCa cells, through activation of the Jagged1/Notch1 pathway, has recently been shown in promoting tumorigenesis [159]. It has been reported that chronic exposure to MSC abets the selection of PCa cells that are resistant to IL-28-induced apoptosis and treatments such as docetaxel, which depends on the MSC secretion of TGF-β1 [160].

## 4. Tumor Stroma from Bone Metastasis

Metastasis requires successive steps. First of all, migratory PCa cells invade blood vessels, survive in the circulation, leak and nest in a secondary metastatic site [161]. This is an inefficient process, with a chance rate of only 0.01% of tumor cells achieving this complete process [162]. PCa predominantly forms bone metastases [163], which are known to cause severe symptoms such as vertebral fractures and/or spinal cord compression. The PCa bone tropism is probably due to the SDF-1/CXCR4 pathway. In fact, an experimental mouse model demonstrated that endothelial cells and osteoblasts in the bone marrow release CXCL12, which attracts PCa cells expressing the CXCR4 receptor [164]. The fact that PCa cells, by expressing α2β1 integrin, show preferential adhesion to collagen is also relevant [165]. Consequently, high collagen levels may also contribute to bone tropism toward the bone matrix [166]. In this context, the CAFs involved in deposition of the ECM components, such as collagen, fibronectin and tenascin, may contribute to critical protein interactions within the metastatic niche [167,168]. Interestingly, tenascin, which is absent in adult bones, may be re-expressed during PCa bone metastasis, and metastatic PCa cells interact with tenascin through α9β1 integrin [168]. In addition, it has been reported that tenascin detected in high levels in the circulation from PCa patients previously to a radical prostatectomy could contribute efficiently to predicting BCR-free survival [169].

## 5. Emergent Role of the Extracellular Vesicles in the Intercellular Signaling from Tumor Microenvironment

**Figure 4:**
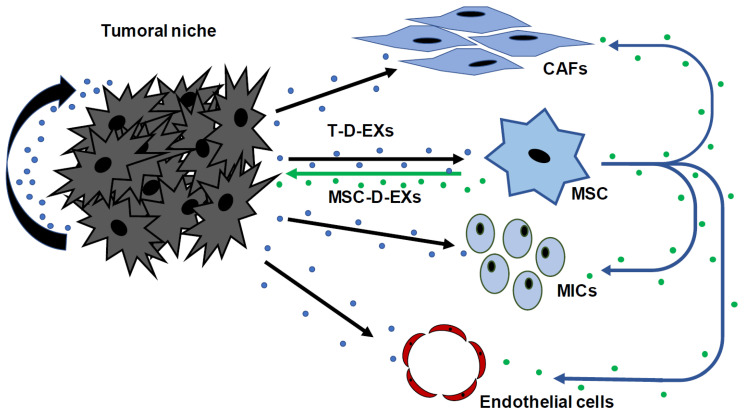
Schematic representation of paracrine interactions through exosomes among different cell types from prostate carcinomas. T-D-EXs: tumor-derived exosomes; MSC-D-EXs: mesenchymal stem cell-derived exosomes; CAFs: cancer-associated fibroblasts; MICs: mononuclear inflammatory cells.

Extracellular vesicles (EVs) are responsible for a concrete nano-communication system among the different cell types of the tumor (Figure 4). They can be sorted into three different categories based on their size: apoptotic bodies (1000–5000 nm), microvesicles (500–1000 nm in diameter) and exosomes (30–150 nm) [170]. Exosomes, which originate in the endocytic compartment, withhold, at least partially, the content of the parent cell [171], such as cytokines; growth factors and nucleic acids (mRNA, miRNA and DNA), among others [172].

EVs acquired special interest from the clinical use of liquid biopsies to explore circulating tumor cell (CTC)-derived products [173]. In addition, the presence of two PCa cancer RNA biomarkers in EVs isolated from urine was demonstrated: TMPRSS2:ERG and PCA3 [174]. A more recent study supported the interest of urine EVs for the diagnosis of PCa, especially high-grade cancer [175]. Plasma and serum EVs have also been found as potential biomarkers for a PCa diagnosis [176]. In addition, tumor-derived EVs were found significantly higher in plasma from patients with CRPC and associated with a dimmer chance of survival [177]. On the other hand, it was reported that the presence of EVs containing specific miRNAs predict radiation therapy efficacy [178] or biochemical recurrence after radical prostatectomy [179].

PCa EVs also promote a tumor-supportive environment by inducing reprogramming of the stroma [180,181]. It has been proven that tumor-derived exosomes (T-D-EXs) induce changes in MSCs, both phenotypic and functional, which might wield profound effects on tumor growth [182] and epigenetic changes that can be promoted by the genetic cargo of T-D-EXs [183]. The mechanism of which T-D-EXs impact MSCs is not known, and it has not been elucidated yet if a protein transfer is enough or if nucleic acids and transcription factors are required [184]. It has been described that T-D-EXs from chronic lymphocytic leukemia, breast cancer or PCa can stimulate MSC migration to the tumor site [185] and MSC differentiation into myofibroblasts, which causes the overexpression of αSMA [186]. Dai J et al. reported a prime example of said interactions, witnessing that PCa-derived EVs promote bone metastasis through the EV-mediated transfer of pyruvate kinase M2 from PCa cancer cells into bone marrow stromal cells [187].

EVs have also been found to play a key role in the paracrine communication between PCa cancer cells and CAFs [188]. Atypically large EVs released by PCa cells further enhance the migration of CAFs by the intercellular transmission of functional miRNA such as miR-1227 [189]. It was also shown that PCa EVs induce a pro-tumorigenic phenotype in fibroblasts via TGF-β, which promotes angiogenesis and tumor growth [190,191]. Furthermore, CAFs produce exosomes containing microRNA-409, which is known to inhibit the translation of tumor-suppressor genes, hence promoting EMT and tumor invasiveness [192]. They have also been shown to induce the migration and invasion of PCa cells via the CX3CL1-CX3CR1 pathway [193]. CAF-derived EVs contain amino acids and lipids that may be utilized by cancer cells under nutrient deprivation conditions [194].

It was also reported that EVs are responsible for reciprocal interactions between both PCa and immune cells. Thus, PCa-derived EVs facilitate immune evasion by downregulating natural killer and CD8+ T cells [195]. In addition, the interaction between TAMs and the EV-mediated transfer of miR-95 is known to promote PCa progression [196]. On the other hand, MSC-derived exosomes arise special interest in the context of intercellular communication. Under physiological conditions, MSCs behave as a munificent source of exosomes [151], seemingly responsible for numerous functions that are broadly attributed to MSCs, such as their influence on adjacent stromal cells [197,198]. First and foremost, MSC-derived exosomes are able to interact with a wide variety of cell types in order to assure they appropriately uphold the tumor growth (Figure 4). MSC-derived exosomes transport a variety of molecules and genes comprising more than 850 gene products and 150 miRNAs [199,200], which allow them to impact on different cellular responses in several cells [201]. Remarkably, MSCs are receptors of signals generated by the tumor and, in turn, accomplished producers of their own exosomes. Therefore, there is a horizontal transfer of information carried out by exosomes to neighboring cells that molds the physiological environment to one supporting tumor survival [182].

## 6. Tumor Stroma and Therapeutic Opportunities

Several studies have shown that the tumor stroma, although being morphologically abnormal, is genetically intact and stable [202,203,204]. This suggests that stromal cells may be more susceptible to therapeutic intervention than the genetically unstable tumor epithelium.

The concept that targeting the stroma is a viable therapeutic option has been widely consolidated by the available strategies targeting angiogenic cells in clinical trials on patients with advanced breast cancer [205]. Cancer therapies should focus on progressively disrupting the dynamic interaction between neoplasm cells and the tumor milieu by aiming at metabolic deregulation and inflammation so the tissue homeostasis will be partially restored and the immune cancer kill switch turned on. However, this therapeutic approach would require a deeper understanding of the interactions among the cancer cells, the TME and the immune system, given the adaptive complexity of said communications. For instance, based on the knowledge that the interaction between HGF secreted by the stroma cells with its c-Met receptor located in the epithelium must occur for PCa cells to become migratory, it was shown that resveratrol inhibits HGF-mediated interactions between the stroma and the epithelium and suppresses epithelial PCa cell migration by attenuating EMT [206].

### 6.1. Inhibing CAFs

Considering the protumor functions exerted by CAFs, we could devise therapeutic strategies, such as reprogramming CAFs into normal fibroblasts or by blocking signaling pathways involved in the crosstalk between CAFs and cancer cells [64,207]. In addition, compared to cancer cells, CAFs are genetically more stable and have fewer chances of developing drug resistance, thus representing a therapeutic target less likely to develop chemoresistance [208,209]. Consequently, diverse strategies could be developed associated with said CAFs, such as targeting their capacity to use mechanical forces on the basal membrane [210] or induce lactate reduction in order to drive the TME towards a less inflamed state so the immune system can perform an effective intervention. This happens, in part, because of the possible dysregulation of the RTK, PI3K and MAPK signaling pathways, which can be the first promoters of upregulated glycolysis in neoplasm cells. The subsequent increase of lactate production into the TME will lead to its acidification and the ensuing activation of TGF-β [211], which prompts the recruitment and transformation of CAFs. Far from being purely hypothetical, new agents blocking CAF protumor activity have already undergone preclinical and even clinical evaluations [212,213]. Regarding PCa, it has been shown that YAP1 can convert normal fibroblasts into CAFs in this carcinoma microenvironment. Therefore, silencing YAP1 in tumor stromal cells can effectively inhibit tumor growth [53]. It has been also demonstrated that endo-, phyto- and synthetic cannabinoid treatments are able to simultaneously strike PCa cells and CAFs [214]. In addition, it was suggested that, considering that CAF-secreted exosomal miR-423-5p promoted chemotherapy resistance in PCa cells by targeting GREM2 through the TGF-β pathway, the inhibition of miR-423-5p might enhance the drug sensitivity of PCa [82].

### 6.2. Immunotherapy

Several clinical trials on the effectiveness of inhibitors of cytokine receptors and/or neutralizing antibodies to avoid the exposure to inflammatory factors that contribute to tumor progression have been conducted [215,216]. Among the most considered immune inhibitors were those ones against programmed cell death protein 1 (PD-1) and cytotoxic T-lymphocyte antigen-4 (CTLA-4) [217,218]. The immunotherapies showed durable clinical responses in tumors such as renal cell cancer and melanoma [219,220]. However, these therapeutic potentialities have not yet been confirmed in PCa [218].

The TME of PCa is highly immunosuppressive due the actions of the immune cells (regulatory T cells, TAMs and MDSCs) [218]. This immunosuppressive effect mediated by cytokines (TGF-β, adenosine, IL-6, IL-8, IL-10 and VEGF); prostaglandin E2 and programmed death-ligand 1 (PD-L1) with programmed cell death protein 1 (PD-1) [221], as well as the secretion of adenosine via prostatic acid phosphatase. However, most of the trials in PCa have targeted a single immunosuppressive mechanism, so the clinical efficacy is likely to be limited. The use of combination therapies to avoid multiple mechanisms of resistance should be considered [218]. Furthermore, there are data indicating a relationship between ADT and the immunological antitumor response by inducing immune cell infiltration and increasing the sensitivity of tumor cells to immune-mediated lysis. In addition, mice receiving a combination of enzalutamide treatment with a cancer vaccine had a significantly increased overall survival [222,223,224]. In this sense, many clinical trials have shown an increase of the antitumor effectiveness of immunotherapies when combined with ADT [225,226,227]. In fact, it was reported that immune-related genes (JUNB, SOCS3 and ZFP36) may have a key role in the ADT immune remodeling process in PCa, which impact the prognosis [228]. Consequently, it is essential to comprehensively describe the PCa immune microenvironment in order to facilitate identifying suitable patients to undergo immunotherapy. In this sense, certain alterations such as dysfunctional DDR, CDK12 alterations or microsatellite instability have been identified as advantageously responsive to immunotherapy in PCa [229,230,231].

### 6.3. MSC as New Therapeutic Strategy

Non-associated tumor MSCs are widely distributed among tissues, and they display a key role in homeostasis [232,233]. It is possible to conceive an antitumor alternative based on MSCs if we were to take into consideration the protumor or antitumor effects dependent from their tissular origin and tumor lineage [234,235]. For example, MSCs of reproductive sources seem to have an antitumor effect on specific carcinomas [234,236]. It was even reported that MSCs from uterine cervix origin display not only anticancer effects against triple-negative breast cancer cells but also against protumor stromal cells, such CAFs and cancer-associated macrophages [237,238].

Based on the mentioned precedents, and the known tropism for tumors MSCs exhibit, the idea of tracking down a specific type of MSC with antitumor effects against PCa is neither utopian or far-fetched [239]. MSCs may be developed as vehicles for drug delivery. For example, MSCs may deliver oncolytic viral loads into tumors [240,241]. MSCs have also been genetically manipulated to express immunomodulatory cytokines, which can promote cancer cell killing effects. MSCs genetically modified to produce IFN-β induce significant antiproliferative effects in metastatic PCa preclinical models [242]. In addition, MSCs have been genetically manipulated to express specific enzymes, as aforementioned, such as herpes simplex virus-thymidine kinase (HSV-TK) or cytosine deaminase, which can convert administrated prodrugs, such as fluorouracil and ganciclovir, into active cytotoxic agents. Therefore, this strategy may increase the antitumor activity of chemotherapy and minimize the systemic toxicity, as demonstrated in experimental models of PCa [243,244].

However, cell-based therapies have brought to the forefront several safety issues related to the transplantation of breeding living cells, including, but not limited to, immunological mismatch, the formation of emboli, the possible chance of MSC entering into senescence and even tumorigenicity. Nonetheless, scientific data show that the beneficial effects of MSC endure through the secretion of paracrine factors (cytokines and growth factors) and EVs. Due to the anti-inflammatory, antioxidative stress, regenerative, angiogenic and antiapoptotic capabilities from these components, MSC secretome should be studied as a promising candidate for new medical biotechnology [245]. Furthermore, the usage of EVs of the MSC secretome, unlike cellular therapies, can be better assessed in terms of the safety, efficiency and dosage and in a very dissimilar way to conventional therapeutic agents. Secretome endures storage without cryopreservative agents and their potential toxicity. The use of secretome-derived products has proven to be cheaper and more feasible for clinical use, since the employment of the secretome is nowhere near as expensive, in both time and capital, as expanding and maintaining clonal cell lines. Needless to say, secretomes for therapies, such as the conditioned medium of exosomes, could be prepared in advance and be available for treatments when required [246].

Interestingly, it is estimated that the coalition of paracrine factors, summarized as secretomes, are responsible for up to 80% of the therapeutic impact of MSCs. It has been conveyed that MSCs secrete high amounts of tumor growth-inhibiting cytokines, such as CXCL10, IL-12, IFN-α, IFN-β, IFN-γ, DKK-1/3, latency-associated peptide (LAP), TNF superfamily member 14 (TNFSF14), also known as LIGHT, TRAIL (Tumor Necrosis Factor-Related Apoptosis-Inducing Ligand) and the Fms-related tyrosine kinase 3 (FLT-3) ligand. The antitumor effect of MSCs has also been reported as being partly subservient to TIMP-1 and TIMP-2 activity, both abundant in the secretome. This may be due to MMP inhibition, these enzymes being related to the migration and invasion of cancer cells [234]. Commonly, it is assumed that MSC-derived EVs render akin functions to their parent cells [247], some of which may also be antitumor effects [234]. This is the case, for example, of the AD-MSC-EVs, which evinced PCa growth-inhibiting behavior [248]. MSC-EVs arise further interest for oncological therapy due to their tumor tropism. It is also known that cancer cells internalize a higher amount of exosomes compared with normal cells [249,250]. On the other hand, exosomes can be loaded with anticancer particles (for example, cytotoxic chemotherapy agents, small interfering RNA (siRNAs) or miRNAs) using different techniques, such as incubation, by the transfection of exosome-producing cells or by chemical transfection electroporation [251].

In summary, using MSCs as anticancer therapy might turn out to be an interesting strategy, provided we conduct the appropriate experimental models to explore the mechanisms. However, we need to resolve several aspects, such as obtaining an optimal MSC secretome product for PCa treatment, ensuring their standardization and mass in vitro production in bioreactors and the use of functional assays to test the obtained biological products.

## 7. Conclusions and Future Perspectives

The two main unresolved concerns about PCa are the absence of more precise prognostic factors to identify patients at risk of metastasis and the need of more effective treatments for them. Most researchers have focused on the characteristics of PCa cells rather than on the stromal components. Due to this, the stromal component in PCa has not been studied as much as in breast cancer.

The stroma of PCa offers many possibilities for future research. The dynamic aspects of this structure may reflect the complex cellular inter-signaling of PCa and may even be interconnected with mechanisms through which lifestyles can considerably influence prostate carcinogenesis. In this sense, for example, it has been described that obesity was affiliated with shorter telomeres in PCa-associated stromal cells, which was correlated with an increased risk of PCa fatal outcome [252,253]. In this line, more recently, it was reported that, among men with the aggressive disease (Gleason ≥ 4 + 3 and stage > T2), these ones with obesity had three-fold increased odds of shorted telomeres in prostate stromal cells when compared to normal weight men. Therefore, it was suggested that telomere shortening in prostate stromal cells may be one mechanism through which someone’s lifestyle influences a dire prostate carcinogenesis [254].

Recent studies also showed interest in integrating panels of PCa tumor stromal markers that, as with the expression of CD31 (vascular marker), alpha smooth muscle actin (αSMA) and PR expression ratio between the PCa stroma and prostate normal tissue stroma, play a crucial role in the onset and progression of PCa [255]. In addition, new studies are demonstrating the importance of considering mathematical computational models that integrate the classic clinicopathological factors derived from a PCa epithelium tumor with recently gathered data from the functional biology of the stroma, such as single-cell RNA-Seq, whole-exosome sequencing, proteomic and metabolomic methods [256,257,258]. Thus, the stroma could be a contributing factor in discriminating against PCa that differ widely in their prognoses. Nevertheless, further research on the molecular mechanisms of tumor–stroma interactions is still needed to develop novel therapeutics based on targeting stromal-derived protumor activities in PCa [207].
